# A Sequel to Novocain Injection

**Published:** 1913-03-15

**Authors:** Simonds Gooding, F. Ledger Etheridge


### A SEQUEL TO NOVOCAIN INJECTION.

BY SIMONDS GOODING, M.A., M.D., CANTAB., AND F. LEDGER ETHERIDGE, L.D.S.

E. P., an unmarried girl aged 20, in domestic service, at 5 p.m. on November 6 was injected with 40m. of a 2 per cent. solution of novocain to have three upper incisors extracted. The operation was successfully performed, the patient stating afterwards that she felt perfectly well. She went home to supper with her parents, returning later to her mistress's house, where she carried out her usual duties, and went to bed apparently in good health at 10 p.m.—that is, five hours after the administration of the novocain.

The following morning her mistress called her as usual; she was unable to get a reply and allowed her to sleep on. The maid remained unconscious for the next twenty-four hours, and her mistress, becoming alarmed, sent for a doctor, who pronounced her to be suffering from cocaine poisoning. The girl's parents were then communicated with, and at their desire one of us (S. G.) saw her at 11 a.m. on November 8, about forty-two hours after the extraction of the teeth.

The patient was a well-developed muscular girl who rather presented the appearance of a case of cerebral irritation. Her face was slightly flushed, but respiration, pulse, and temperature were normal. She resented any interference. The jaw was tightly clenched; she refused to attempt to swallow or to take heed of what was said to her. She kept her eyes firmly closed; on opening them the pupils were equal and dilated, they reacted to light. The deep and superficial reflexes were normal. The bladder was found to be nearly empty, although according to the history she had been in an unconscious co[illegible]tion about thirty-eight hours. The catamenia had just commenced. As every effort to rouse her was useless, she was ordered 2m of croton oil, to be given in butter. By 6 p.m. she had considerably improved; though still drowsy she would answer questions. The bowels had acted as the result of the croton oil, and for that purpose she had been able to get out of bed. She had taken milk. Her jaw was no longer clinched, and she was now awake. Two hours later she went to sleep and slept until the morning, when again it was found to be impossible to rouse her, and at 11 a.m. on November 9 her condition was exactly the same as on the previous day. The croton oil was repeated with the same effect; but when the patient got out for the bowels to act she was not allowed to go back to bed, but was assisted to dress, and at 6 p.m. she opened the door perfectly recovered, and has remained well since.

Adding further clinical particulars, with historical notes on novocain, the authors remark:

The curious relapse and ultimate instantaneous and complete recovery, combined with the other points in the case, all point to it being a case of feigned coma in a hysterical subject rather than one of toxaemia due to a narcotic poison. —*British Medical Journal*, December 7, 1912.

